# Thermoresistive Strain Sensor and Positioning Method for Roll-to-Roll Processes

**DOI:** 10.3390/s140508082

**Published:** 2014-05-05

**Authors:** Kuan-Hsun Liao, Cheng-Yao Lo

**Affiliations:** 1 Institute of NanoEngineering and MicroSystems, National Tsing Hua University, No. 101, Section 2, Kuang Fu Road, Hsinchu 30013, Taiwan; E-Mail: s9935804@m99.nthu.edu.tw; 2 Department of Power Mechanical Engineering, National Tsing Hua University, No. 101, Section 2, Kuang Fu Road, Hsinchu 30013, Taiwan

**Keywords:** alignment, gauge factor, infrared, Joule heating, polyethylenenaphthalate, registration, roll-to-roll, sensor, strain, thermoresistive

## Abstract

This study uses the Joule heating effect-generated temperature difference to monitor in real-time and localize both compressive and tensile strains for the polymer substrates used in the roll-to-roll process. A serpentine gold (Au) line was patterned on a polyethylenenaphthalate (PEN) substrate to form the strain sensor based on thermoresistive behavior. This strain sensor was then subjected to either current or voltage to induce the Joule heating effect on the Au resistor. An infrared (IR) detector was used to monitor the strain-induced temperature difference on the Au and PEN surfaces and the minimal detectable bending radius was 0.9 mm with a gauge factor (GF) of 1.46. The proposed design eliminates the judgment ambiguity from conventional resistive strain sensors where resistance is the only physical quantity monitored. This study precisely and successfully indicated the local strain quantitatively and qualitatively with complete simulations and measurements.

## 1. Introduction

Strain sensors are widely used to evaluate material and structural failures or weak points. Because strains generated in the micrometer range are difficult to visualize in real-time using the bare eyes, they must be detected based on physical quantities read out by humans or automatic systems. Light-based optical strain detection solutions usually take advantage of unique optical effects, such as the Fabry-Pérot interference [1,2], the Moiré effect [3,4], Bragg diffraction [5,6], or pattern recognition [7], to visualize strain with visible colors or non-visible signals. In the same time, sound waves are also used to monitor the density of materials, which in turn reflect strains embedded inside. The surface acoustic wave (SAW) system [8] is one of the strain sensors that take advantage of sound waves. Other novel strain sensors based on X-rays [9] and radio waves [10] were not considered and compared here because of their complicated setup and higher power consumption.

In addition to optical and acoustical strain sensors, other physical quantities like resistance [11,12], capacitance [13,14], inductance [15], and magnetic field [16] are also used to measure strains. Among these options resistive strain sensors are widely used because of their simple detection mechanism. Resistance varies in proportion to the resistor length change, and the material strain exhibits a relationship with the resistance. Corrections are occasionally necessary because the thickness and width of a resistor also change with its change of length. Table 1 lists conventional resistive strain sensors with their characteristics, where the sensitivity represents the smallest detectable strain in the corresponding system, and the negative and positive signs represent compression and tension in the strain detection range, respectively. The definition and calculation of the gauge factor will be described later.

**Figure 1.**
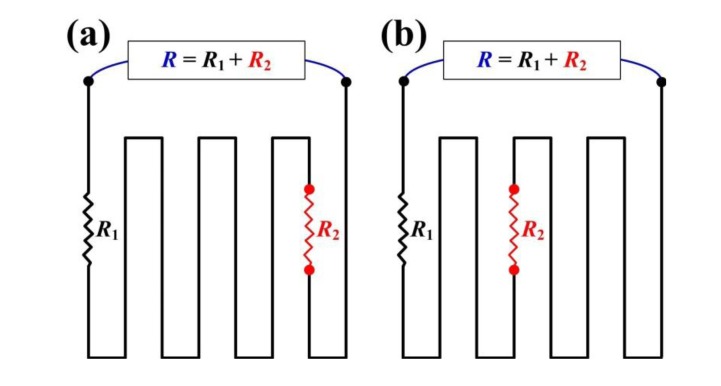
Explanations of the ambiguous judgments from a conventional resistive strain sensor, on which combinational resistance cannot identify local behaviors. The superimposed total resistance (*R*) in (**a**) and (**b**) cannot reflect different local resistances (*R*_1_ and *R*_2_) and their distributions.

Nevertheless, these resistive strain sensors use only one physical quantity–resistance–and lack application variety and location accuracy. The output signals from a resistive strain sensor only reflect overall performance, and overlook local details. For example, when a target object has one small-value-tension connected in series to another large-value-compression, the monitored resistance returns only a superimposed result of a small-value-compression. Similar ambiguity also appears for resistors for strain sensor applications connected in parallel. This overlooked result is misleading and leads to incorrect judgments (Figure 1).

From the roll-to-roll (R2R) mass production viewpoint, a methodology that detects substrate strains in real-time is necessary, otherwise the tens to thousands of meter-long substrates shift during winding and unwinding. This represents a critical manufacturing issue for flexible electronic devices, where micrometer or nanometer registration (alignment) shifts can result in device malfunction or failure. This pattern shift will be further worsened if a multiple layer stack is used for the device. Previous work proposed a detection methodology for the starring and telescoping effect in the R2R system [21]. However, it requires external strain sensors with wound rolls before the uneven stress distribution can be returned to the controller and then adjusted by mechanical parts. Although it solved off-line the winding/unwinding shifts, it neither handles micrometer strains nor corrects the shifts in real-time. Furthermore, the external strain sensor arrangement is a manual task, which cannot be performed automatically. To solve these issues, this study proposes an advanced resistive strain sensor that introduces a second physical quantity of heat to localize the strain quantitatively, qualitatively, and automatically.

## 2. Design

**Figure 2.**
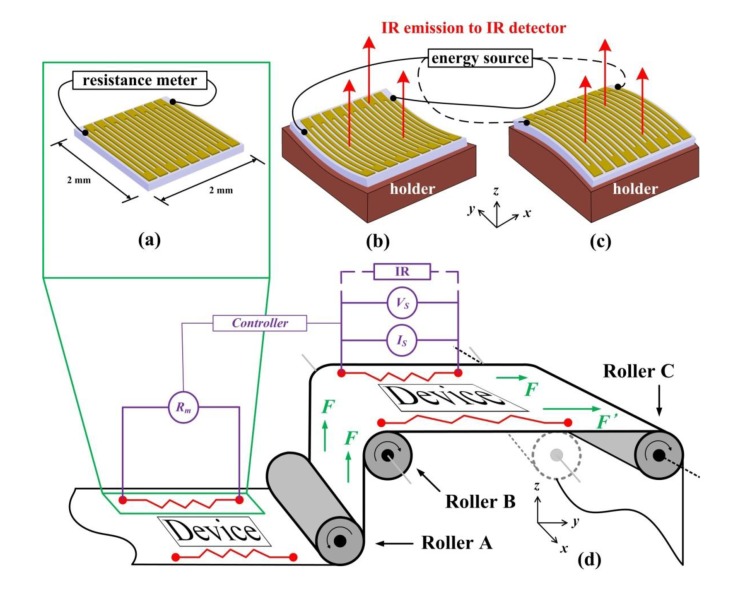
(**a**) The proposed strain sensor consists of a serpentine Au resistor on the polyethylenenaphthalate (PEN) substrate with a voltage or a current source. The detection demonstration system has an infrared (IR) detector above the (**b**) concave and (**c**) convex holder underneath the strain sensor to measure the compression and the tension cases, respectively. (**d**) The roll-to-roll manufacturing process with the proposed method. A side-to-side balanced force (*F* and *F′*) on the roll becomes unbalanced (*F* and *F′*) with an off-axis roller (Roller C). The roller in (**d**) in dashed line is the correct and expected location for the Roller C. Extra resistance meter (*R*_m_) and controllers help to adjust the voltage source (*V*_s_) and current source (*I*_s_) to eliminate initial resistance variations.

Figure 2 shows the proposed device and the detection system setup. A serpentine metal resistor was patterned by lithography on the polymer substrate with large routing pads at both ends. A 125 μm thick polyethylenenaphthalate (PEN; Teijin DuPont, Q65F, Wilmington, DE, USA) was chosen as the substrate to reflect the reality of a roll-to-roll manufacturing system for flexible electronic device applications, which generally faces mechanical accuracy (strain) issues (Figure 2d). Gold (Au) of thickness of 10 nm was chosen as the resistor because of its sensitive electrical properties and good thermal conductivity during application and measurement. The Au resistor was directly patterned on the PEN by photolithography without adhesion concerns [22] in this study. Other polymer substrates and metal resistors can also be used similarly to this proposed scenario.

## 3. Principle

During operation, a current (*I*) or voltage (*V*) is applied to the Au resistor with resistance (*R*), which generates thermal energy (heat, *P*) because of the Joule heating effect. The thermal energy is positively (*P* = *I*^2^*R*) and negatively (*P* = *V*^2^/*R*) related to the resistance under a fixed current or voltage, respectively. When the PEN substrate and the Au resistor above it change their dimensions, the resistance changes accordingly. With the aforementioned Joule heating effect, users can obtain deformation and strain information by understanding the infrared (IR) emissions from the Au resistors located on both sides of the substrate.

The sensitive electrical behavior of 10-nm-thick Au enhanced the temperature change because its resistance highly reflects the dimensional change. The operation of this proposed methodology uses the resistance change (Δ*R*) instead of absolute resistance value (*R*), thus slight initial resistance variations caused by the patterning process before Roller A do not contribute to the temperature change (Δ*T*). Use of a well-developed and commercially available resistance meter (*R*_m_) with controllers to adjust voltage source (*V*_s_) or current source (*I*_s_) in Figure 2d further eliminate the initial resistance variation concern, which is outside the scope of this study.

**Figure 3.**
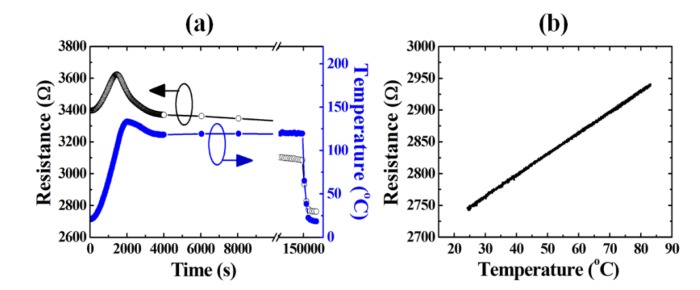
(**a**) The preliminary thermal treatment at 120 °C for 36 h assured a metal sintering-ineffective Au resistor during application. After the preliminary thermal treatment, the resistance reduced from over 3400 Ω to less than 2700 Ω, making the resistance thermally insensitive during Joule heating. (**b**) The linear thermal conductivity of the preliminary thermal treated Au resistor on PEN. The data points in (**b**) represent one temperature cycle (one heat up line and one cool down line) and the two lines are not distinguishable.

On the other hand, the Joule heating effect also gradually reduces the resistance of Au because of metal sintering [23]. In order to decouple the influence of the metal sintering from the strain measurement, a preliminary thermal treatment (120 °C, 36 h) was applied to the Au/PEN structure to greatly reduce the resistance of Au and to generate a stable, metal sintering-ineffective Au (Figure 3a). The linear thermal conductivity of the Au resistor after preliminary thermal sintering assured a linear response between the resistance change and its Joule heating temperature change (Δ*T*, Figure 3b).

For flexible electronic device structures, extra protections (barriers) are required for reliability enhancement. This study proposes a strain detection methodology based on resistance and infrared emission changes, and does not apply extra protection layer on top of the Au resistor.

## 4. Simulation

This study used a mechanical-electrical-thermal coupling model for a simulation using the commercial software COMSOL (version 4.0, COMSOL Inc., Burlington, MA, USA). A full-size model was prepared for the simulation by repeating a single straight Au resistor with 50 μm width, 50 μm space, and 1,500 μm length 18 times with routing pads at two ends (Figure 2a). The routing pads had a larger dimension (150 μm wide and 200 μm long) in the *y*-direction to ensure that the pattern change perpendicular to the loading direction (the *x*-direction) has a negligible influence on the resistance. Because the thermal conductivity of the PEN is far smaller than that of the Au [24,25], this simulation assumed that the heat transfers mainly through the Au/air interface (the upward heat). Furthermore, when the small portion of heat transfers from the Au to the PEN substrate (the downward heat), it transfers to the surrounding PEN substrate in the ±*x*-, ±*y*-, and –*z*-directions sequentially.

**Figure 4.**
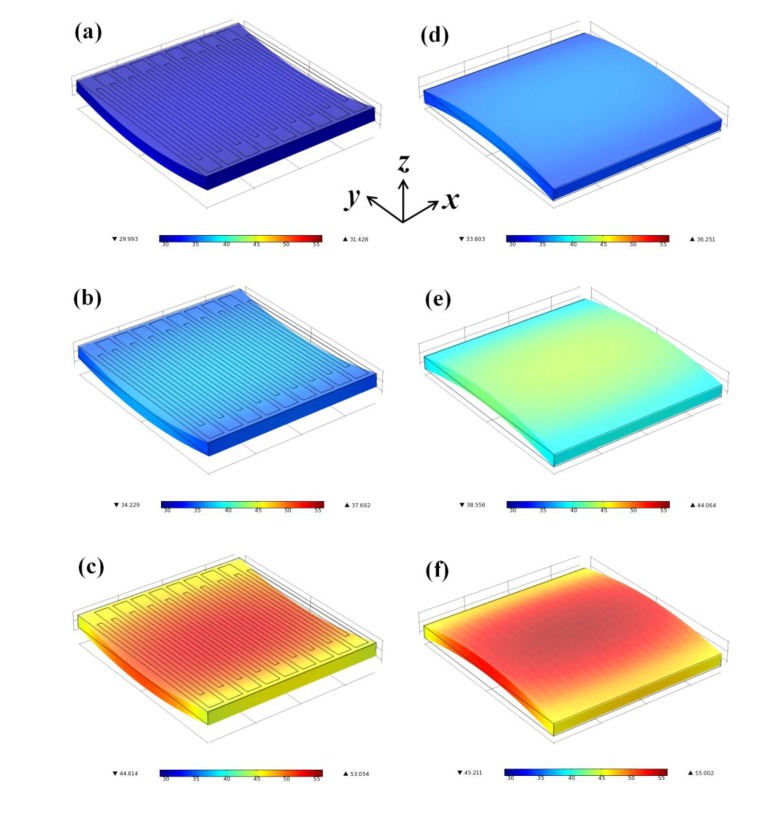
The simulation results for *I* = (**a**) 1 mA, (**b**) 2 mA, and (**c**) 3 mA compression cases, and *V* = (**d**) 6 V, (**e**) 9 V and (**f**) 12 V tension cases. All legends are based on the same settings from 29 (blue on the left) to 56 °C (red on the right). The bending radii for all cases were 25 mm.

Figure 4a–c shows the simulated temperature results for the compression case with various bending radii under fixed current (*I*) application. The heat generated with *P* = *I*^2^*R* distributes across the strain sensor but only accumulates at the center because the heat at the strain sensor edges transfers to the surrounding PEN substrate as mentioned before. Consequently, the temperature distribution on the surface of the strain sensor has its highest value at the center and gradually decreases outwards.

Figure 4d–f shows the simulated temperature results for the tension case with various bending radii under fixed voltage (*V*) supply with *P* = *V*^2^/*R*. Like the temperature distribution of the compression case, the temperature has its highest value at the center and its lowest value at the edge of the strain sensor.

## 5. Results and Discussion

Figure 2b,c illustrates the measurement setup. An IR scope (InfraScope II, QFI Corporation, Vista, CA, USA) was used to monitor the IR emissions from the Au resistor, producing a temperature distribution map during measurement. The environmental temperature was kept at 20 °C for all cases and the resulting temperature map not only reflects the overall average temperature, but also indicates the temperatures at individual points. Owing to the current holder limitations, bending radii (*r*) between 25 and 200 mm were examined. The strains calculated from the sensor dimensions and their bending radii [26] were between 0.065% (*r* = 25 mm) and 0.526% (*r* = 200 mm) for both the compression and the tension cases, which satisfy the reasonable mechanical accuracy requirement of the rollers in the R2R system with substrate side-to-side width (the *x*-direction) of 100–300 mm (Figure 2d).

### 5.1. Compression

**Figure 5.**
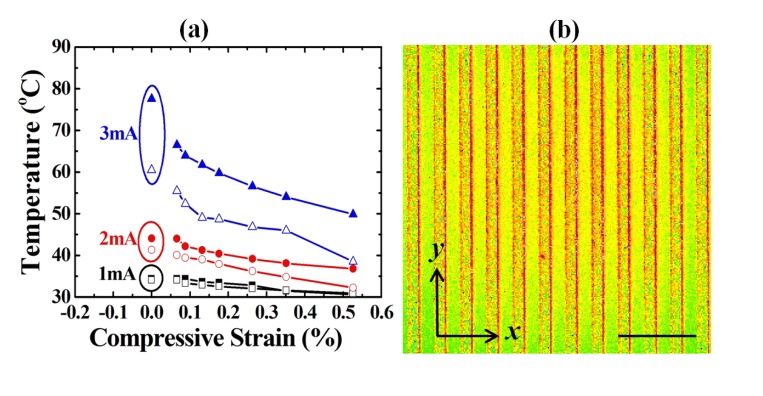
(**a**) The temperature response of various compression bending radii with various applied currents. (**b**) The temperature map of an *r* = 150 mm at *I*= 3 mA (scale bar of 300 μm). Solid and hollow symbols in (**a**) represent simulation and measurement data points, respectively.

Figure 5a shows the relationship between the strains and the measured temperatures of the compression cases. Figure 5b shows the temperature map of an *r* = 150 mm compression case. The proposed methodology distinguished the bending radii from 25 to 200 mm with the reference of a flat surface point (*r* = infinite, strain ε = 0%). The difference between the simulated and measured data results from the variations of simulation parameters (the convection heat transfer coefficient, *h*, and the thermal conductivity, *k*) [27], which depend on the metal sintering-related grain conditions in thin film instead of in bulk material. This difference was negligible under small currents and was enhanced under large current supply. However, the measured data successfully followed the simulation trend, which represented its eligibility for strain prediction from the relative temperature (resistance) changes, instead of from the absolute temperature (resistance) values. The proposed methodology only compared the resistive change (Δ*R*) and both the simulations and the experiment results showed the same Δ*R* and thus, the same Δ*T*.

### 5.2. Tension

**Figure 6.**
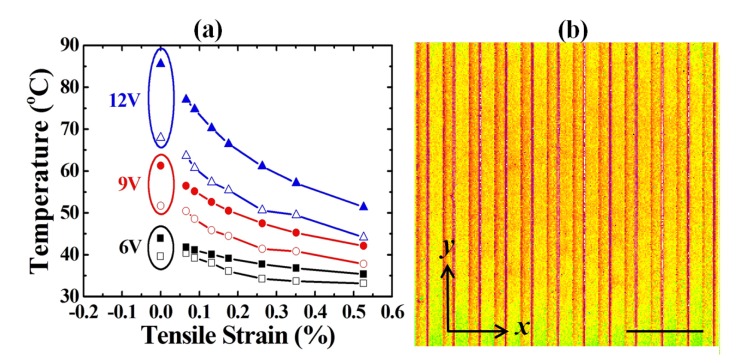
(**a**) The temperature response of various tension bending radii with various applied voltages. (**b**) The temperature map of an *r* = 150 mm at *V* = 12 V (scale bar of 300 μm). Solid and hollow symbols in (**a**) represent simulation and measurement data points, respectively.

Figure 6a shows the relationship between the strains and the measured temperatures of the tension cases. The trend and sensitivity of the tension case are similar to those of the compression case. Figure 6b shows the temperature map of an *r* = 150 mm tension case.

### 5.3. Roll-to-Roll Off-Axis Detection

One of the applications of this proposed methodology is the roll-to-roll manufacturing and is illustrated in Figure 2d. A roll-to-roll system usually contains tens of rollers and the flexible polymer substrate is slightly stretched by these rollers to assure flat substrate surfaces during the process. However, the mechanically controlled tension is usually not precisely balanced from roller to roller.

As a result, the polymer substrate is very possibly stretched more on one side than the other side of a roller. The devices made on the polymer substrate are thus deformed by this issue and their performances suffer from these unpredictable stress-induced strains. For a 500 mm-long (in the *y*-direction) substrate between rollers B and C in Figure 2d, a 1% strain in one side along the mechanical direction (the *y*-direction) represents a 5 mm (5000 μm) extension, which is already an unacceptable value in practice for flexible electronic devices of micrometer or nanometer scales. By intentionally differentiating external forces *F* and *F′* in Figure 2d with 1% strain, the results show that the unstressed side (*F*) did not show a distinguishable temperature difference (69.1 °C at 12 V, compared with the control point of 67.9 °C in Figure 6a) while the stressed side with 1% strain side (*F′*) showed an absolute value of 39.8 °C at 12 V. The 39.8 °C represents a 0.97% strain, which falls on the extrapolated part of the trend line in Figure 6a and proves the correctness of this methodology.

### 5.4. Strain Localization

Another major benefit of the proposed methodology is its ability to localize the strain distributed within the sensing area. Figure 1 shows that the superimposed resistances and their changes of the conventional resistive strain sensors are the same. As a result, the resistances in Figure 1 are both *R*, which overlooks the strain distribution and leads to misjudgment. This issue can only be fixed by an array consisting of smaller strain sensors, but this solution also increases the complexity of the circuit routing, pattern design, sensing methodology, and analysis algorithm. Thus, keeping the same strain sensor configuration but introducing a second physical quantity of heat provides a superior detection resolution as proposed by this study.

**Figure 7.**
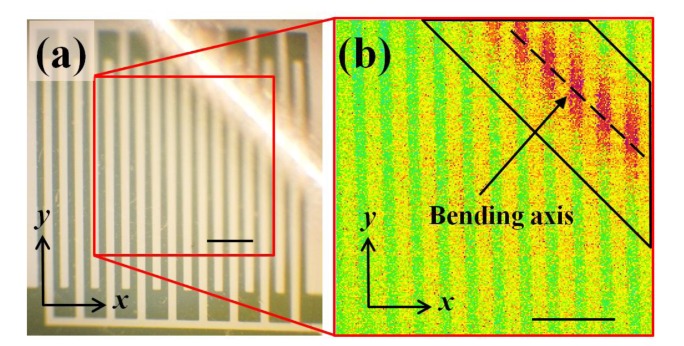
(**a**) An optical microscopic top view of the intentionally folded sample and (**b**) its temperature distribution map. The scale bars in (**a**) and (**b**) are both 300 μm.

As Figure 7a shows, the strain sensor was intentionally folded at one corner. Figure 7b shows the measurement result, which clearly indicates that the heat accumulated at the surrounding areas of the folding mark. Because the resistance changed mainly at the target (trapezoid) area, the temperature change reflected the location of the strain instead of an overlooked overall behavior.

**Figure 8.**
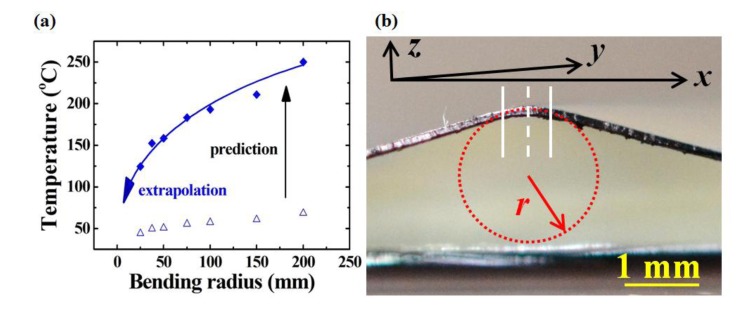
(**a**) The temperature prediction (solid diamond) at 12 V for the resistance in the trapezoid area in Figure 7b based on the experimental results in Figure 6a (hollow triangle). (**b**) The real bending curvature is in good agreement with the extrapolation prediction for the folding mark, where the PEN substrate is 125 μm thick, and the 10-nm-thick Au was unobservable, and therefore, neglected for the bending radius calculation. The dotted line, dashed line, and the solid line in (**b**) respectively represents for the imaginary circle with bending radius *r*, the cross-sectional location of the bending axis in Figure 7b and the cross-sectional location of the outer boundaries of the trapezoid in Figure 7b.

The dashed line in Figure 7b represents the bending axis and the trapezoid extended 250 μm from the bending axis in each direction. According to the heat transfer model [28], this trapezoid covers 83% of the total heat distribution. A smaller trapezoid area contains higher ratio of the high-temperature image pixels but a smaller trapezoid area cannot cover the whole bending area to reflect the reality. In contrary, a larger trapezoid area covers more sample surfaces but a larger trapezoid area also includes less high-temperature image pixels and results in less accuracy. For this study, a 83% coverage was used to evaluate the sensitivity. The trend of the extrapolation line in Figure 8a predicts that the bending radius of the folding mark in Figure 7a was 0.9 mm with the average temperature of 42.6 °C in the trapezoid area in Figure 7b.

The prediction was calculated from the resistance in series with partial contributions (Figure 1). The folding mark was also optically observed (Figure 8b) for comparison, and the bending radius (0.87 mm) agreed with the prediction. This result validated the proposed methodology with a less than 3.3% difference and the gauge factor (GF) of this proposed strain sensor, which is defined as:

$$\text{GF}=\frac{\Delta R/R}{\varepsilon }$$

is 1.46. Here, Δ*R*, *R*, and ε represent the resistance difference between flat and bending conditions, the resistance of the flat condition, and the strain inside the material, respectively.

### 5.5. Surface Morphology and Reliability

The Poisson ratio difference between PEN and Au [29,30] generates different dimensional reductions or extensions under various strains. Metallic Au has better ductility than polymeric PEN and thus extreme volumetric changes of Au on PEN with severe bending potentially produce different surface morphologies. Because the surface morphology of metal reflects its resistance [31,32], it is important to understand the surface roughness and to decouple the dimensional resistance change from morphological influences.

**Figure 9.**
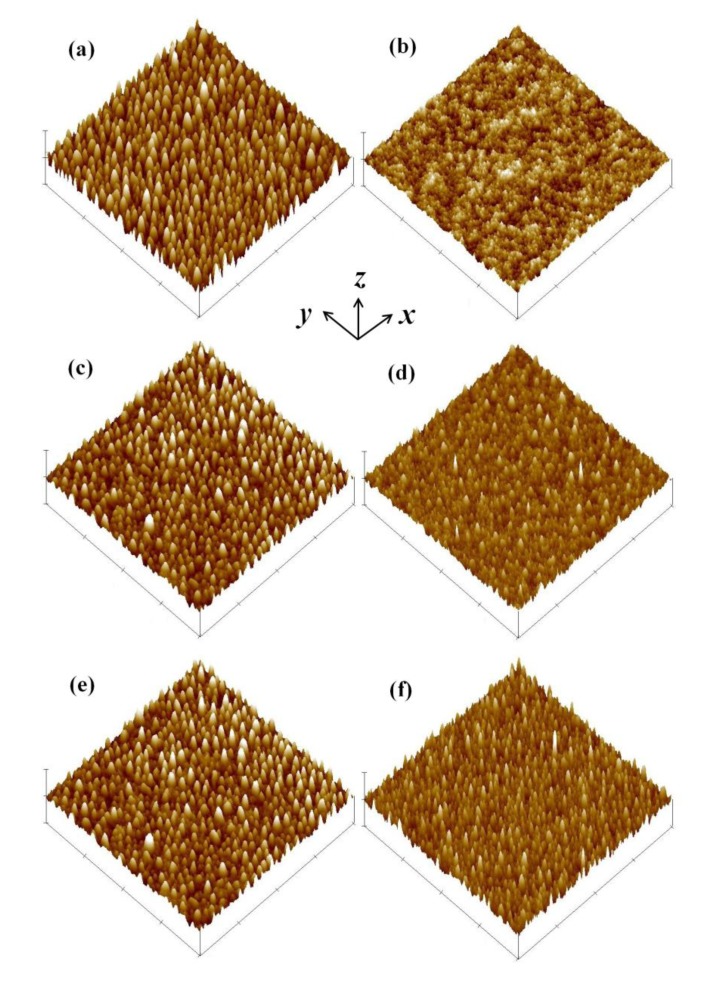
The average surface roughness (R_a_) of the Au resistor for (**a**) before and (**b**) after sintering. The examination area is 2 μm × 2 μm. The R_a_values are in the same level for (**c**) single compression, (**d**) single tension, (**e**) 200-time compression, and (**f**) 200-time tension at *r* = 25 mm. The corresponding *R*_a_ values are (a) 3.84 nm, (b) 1.24 nm, (c) 2.37 nm, (d) 1.61 nm, (e) 2.00 nm, and (f) 1.68 nm, respectively.

Figure 9 shows the surface conditions of Au examined using an atomic force microscope (AFM). This study applied two examination methodologies, single bending and cyclic bending, with various bending radii. After preliminary thermal treatment at 120 °C for 36 h (Figure 9b), the sample shows better surface roughness than before preliminary thermal treated one (Figure 9a). Although samples after compression treatment generally showed worse average surface roughness (*R*_a_) than the tension-treated ones, samples with severe (*r* = 25 mm) treatments (Figure 9c,d) exhibited surface roughness similar to that of the flat case (Figure 9b). The 200-time cyclic bending sample also exhibits the same level of surface roughness, as shown in Figure 9e,f. Because the reported intrinsic surface roughness of thin Au film is less than 10 nm [31,33,34], these results prove that the resistance change comes from the strain, which resulted in either length reduction (from compression) or length extension (from tension), not from the surface morphology. The cyclic bending test further indicates that the strain sensor has good reliability over repeated operations. With these results, this advanced resistive strain sensor functions as predicted.

## 6. Conclusions

This study presents an advanced resistive strain sensor with superior strain localization capability by introducing a second physical quantity of heat. This study also presents a complete analysis based on theory, simulation, experiment, and reliability test. Using temperature (infrared emission) detection, this strain sensor provides a non-contact measurement method. With different applied currents or voltages, the sensitivity of the strain sensor can be changed to suit specific application requirements. By preliminarily patterning this strain sensor on the flexible substrate with a proper energy supply, mechanical stress-induced substrate deformation in a roll-to-roll manufacturing system can be monitored in real-time. This detection methodology requires only low-cost materials and simple processes, eliminates data analysis ambiguity, and supports both compression and tension measurements with localization options. With the help of strain feedback and automatic roller positioning, which is out of the scope of this study, a real-time in-line roller correction can be further realized.

## Figures and Tables

**Table 1.** Specification comparison between different resistive strain sensors.

	**Sensor size**	**Maximum Gauge Factor**	**Detection Range**	**Sensitivity**
Carbon nanotube [17]	7 mm × 10 mm	22.4	−0.006%–0.006%	0.001%
Nickel [18]	20 mm × 60 mm	15	Fixed at 0.03%	0.03%
Gold nanoparticle [19]	Diameter of 5 mm	300	0%–0.55%	0.22%
Graphene [20]	50 mm × 50 mm	0.997	0%–2%	0.167%
This work	2 mm × 2 mm	1.46	−0.526%–0.526%	0.012%
